# Impact of the COVID-19 Pandemic on the Mental Health of College Students in India: Cross-sectional Web-Based Study

**DOI:** 10.2196/28158

**Published:** 2021-09-02

**Authors:** Amar Prashad Chaudhary, Narayan Sah Sonar, Jamuna TR, Moumita Banerjee, Shailesh Yadav

**Affiliations:** 1 Department of Pharmacy Practice Mallige College of Pharmacy Rajiv Gandhi University of Health Sciences Bangalore India; 2 Department of Pharmacology Mallige College of Pharmacy Rajiv Gandhi University of Health Sciences Bangalore India; 3 Department of Pharmaceutics Mallige College of Pharmacy Rajiv Gandhi University of Health Sciences Bangalore India

**Keywords:** anxiety, COVID-19, depression, fear, FCV-19S, GAD-7, mental health, pandemic, PHQ-9, students

## Abstract

**Background:**

The COVID-19 pandemic has created a mental health crisis among college students in India due to lockdown restrictions, overwhelming numbers of COVID-19 cases, financial difficulty, etc. This mental health crisis has led to high degrees of fear, anxiety, and depression among college students.

**Objective:**

The aim of this study is to investigate symptoms of fear, depression, and anxiety due to the COVID-19 pandemic among college students in India.

**Methods:**

This cross-sectional web-based study was conducted using a Google Forms questionnaire. The Google Form included a sociodemographic questionnaire and psychometric scales evaluating the psychological and behavioral impacts of the COVID-19 pandemic. Thus, both qualitative and quantitative analyses were performed in the study.

**Results:**

A total of 324 college students participated in this study, of whom 180 (55.6%) were male and 144 (44.4%) were female. After assessment of the psychometric scales, it was found that of the 324 students, 223 (68.8%) had high fear of COVID-19, 93 (28.7%) had moderate to severe depression, and 167 (51.5%) had mild to severe anxiety. Among the identified risk factors, having a family member who was infected with COVID-19 was significantly associated with anxiety and depression, with P values of .02 and .001, respectively. In addition, the correlations of the Fear of COVID-19 Scale with the Generalized Anxiety Disorder-7 scale and the Patient Health Questionnaire-9 were found to be 0.492 and 0.474, respectively.

**Conclusions:**

This research concludes that there is a very high fear of COVID-19 among students, along with anxiety and depression symptoms. This study also concludes that the Fear of COVID-19 Scale has a moderate positive correlation with the anxiety and depression scales, respectively.

## Introduction

Communicable diseases such as herpes and legionnaires disease in the 1970s, HIV, Ebola, severe acute respiratory syndrome (SARS), and currently, COVID-19, continue to be devastating for global health, creating increasing pressure on people worldwide. The COVID-19 pandemic has kindled a 21st century “viral scare,” following the “microbe panic” of the 20th century. Public health acts such as quarantine, physical distancing, wearing of face masks in public places, and hand hygiene are being executed globally to reduce the spread of infection. Although these measures are efficient to mitigate the pandemic, they may be detrimental to people’s mental health [1].

The transmission of COVID-19 cases in India started to upsurge in the second week of March 2020. Therefore, to prevent community spread of the infection, as in other countries, the Government of India announced a complete lockdown from March 25, 2020, restricting the movement of the entire population of 1.38 billion people in India; this lockdown was initially intended to last 21 days but was extended to May 31, 2020, with conditional relaxation from May 3, 2020 [2]. During this period, all academic institutes were completely closed, and from December, all schools and universities were slowly reopened to resume a normal mode of teaching.

University students, compared to the general public, have been found to be more susceptible to the adverse effects of the quarantine [3]. Mental health disorders are always a topic of concern among youth, and their incidence has been increasing significantly worldwide. According to a World Health Organization report published in 2008, 1 in every 5 adults had experienced mental health disorders in the past year [4]. However, the COVID-19 pandemic triggered an even more rapid upsurge in mental disorders among adults.

An article published in *The Lancet* in February 2020 [5] showed frightening outcomes for people’s mental health even after a quarantine of fewer than 9 days, and these effects could last for up to three years. The very long period of social isolation experienced by students in India during this new epidemic undoubtedly signifies danger, and during this time, the mental health of the students may be affected [6].

Recent studies showed that feelings of anxiety and depressive symptoms, distress, and sleep problems are typical signs of the COVID-19 pandemic. For example, a study conducted by Zhang et al [7] found that 38% of the Chinese population experienced some level of anxiety during the first wave of COVID-19, of whom 16% had severe anxiety; moreover, 49% of the population had depression symptoms, and 14% had severe depression symptoms [7]. Similarly, Wang et al [8] found moderate to severe anxiety, depression, and stress among the Chinese population. Important reasons for these increases in anxiety and depression include the fear of COVID-19 and, more specifically, the fear of becoming infected, along with the loneliness caused by social isolation [6,9]. These findings suggest a negative impact of the COVID-19 pandemic on people’s mental health; therefore, it is urgent to study the scope and source of this impact.

Therefore, the primary objective of this study was to understand the impact of the COVID-19 pandemic on Indian students’ mental health, as in, fear of COVID-19, anxiety, and depression, and to identify risk factors that amplify the magnitude of the psychological effects of COVID-19. The secondary objective of the study was to examine the concurrent validity of the Fear of COVID-19 Scale (FCV-19S) with the Patient Health Questionnaire-9 (PHQ-9) and Generalized Anxiety Disorder-7 scale (GAD-7), respectively.

## Methods

### Study Design and Study Period

The cross-sectional web-based observational study was conducted between November 2020 and February 2021, during which the data collection period was from mid-November 2020 to mid-December 2020. The survey questions and scales were selected based on the available literature, the authors’ knowledge, and the knowledge and experiences of professors and clinicians about the pandemic and its psychological impact. The reporting of the study followed the STROBE (Strengthening the Reporting of Observational Studies in Epidemiology) guidelines [10,11].

### Study Site and Population

For this study, we selected students at the Mallige College of Pharmacy in Bangalore, India, who had access to the internet and used social media.

### Measures

For this study, a specialized web-based form was developed using Google Forms. The form contained two sections, namely, a sociodemographic section and a psychometric scale section; the latter assessed the psychological and behavioral impacts of the COVID-19 pandemic. The scales are as follows:

#### Fear of COVID-19: the FCV-19S

This unidimensional, reliable, and valid self-report scale was recently developed to understand the fear of COVID-19 caused by this pandemic. This scale consists of 7 items that attempt to measure the fear of COVID-19. The responses are recorded on 5-point Likert scales with points ranging from 1 to 5. The higher the score, the greater the fear of COVID-19 among the participants. The initial development of the scale showed robust internal reliability, with a Cronbach alpha of .88 among the Iranian population [12]. A study conducted by Chung-Ying Lin et al [13] to measure invariance issues in the FCV-19S across many countries found that it is a good psychometric instrument to access the fear of COVID-19 [13]. The cutoff scores for this scale are shown in Table 1 [1,14]. In this study, we used the English version of the FCV-19S.

**Table 1:** The score ranges used to evaluate the severity of symptoms of the study participants based on the cutoff scores of the psychometric scales.

Scale	Severity of symptoms (score range)						
	Low fear	High fear	Minimal	Mild	Moderate	Moderately severe	Severe
FCV-19S^a^	0-18	19-35	N/A^b^	N/A	N/A	N/A	N/A
PHQ-9^c^	N/A	N/A	0-4	5-9	10-14	15-19	20-27
GAD-7^d^	N/A	N/A	0-4	5-9	10-14	N/A	15-21

^a^FCV-19S: Fear of COVID-19 Scale.

^b^N/A: not applicable.

^c^PHQ-9: Patient Health Questionnaire-9.

^d^GAD-7: Generalized Anxiety Disorder-7 Scale.

#### Anxiety: the GAD-7

This self-report scale was developed for initially diagnosing generalized anxiety disorder (GAD). The scale consists of 7 items, in which the participant’s responses are recorded on 4-point Likert scales ranging from 0-3. The score of the participant ranges from 0 to 21. The threshold score of 10 has 89% sensitivity and 82% specificity for GAD. The cutoff scores for this scale based on the severity of symptoms are shown in Table 1. In this study, we applied the English version of the GAD-7 [15].

#### Depression: the PHQ-9

This 9-item self-report scale is used to diagnose major depression and subthreshold depression. The participant’s responses are recorded on 4-point Likert scales from 0 to 3. The total score ranges from 0 to 27, and it helps interpret the severity of depression. A score ≥10 signifies moderate to severe depression with significant clinical concern, whereas a score <10 signifies minimal to mild depression. The cutoff scores for this scale based on the severity of symptoms are shown in Table 1. In this study, we applied the English for India version of the PHQ-9 [15].

### Sample Size

The survey study was completed using the Raosoft sample size calculator to capture the appropriate sample size [16]. A minimum of 306 samples was required for a 95% confidence interval and a 5% margin of error for the population distribution of 1500 students at 50% response distribution. Thus, a total of 324 students participated in this web-based study.

### Inclusion Criteria

All students studying for diplomas or degrees, both undergraduate and postgraduate, were included in the study.

### Distribution of the Questionnaire

The Google form was distributed to the students through various social media platforms, such as WhatsApp, Facebook, Messenger, and Telegram. The students were invited to participate in the survey by filling in the Google form without time constraints. Furthermore, the Google feature that limits each respondent to one submission eliminated multiple responses.

### Ethical Considerations

The study was approved by the research review board (Approval MCP/RRB/003/20-21) of the Mallige College of Pharmacy before starting the study. The purpose of the study was explained to the participating students, and they were requested to submit their voluntary consent before participation. All the procedures performed in this study were in adherence to the Declaration of Helsinki of 1964 and its later amendment [17]. Furthermore, this study strictly adhered to the Checklist for Reporting Results of Internet E-Surveys (CHERRIES) guidelines [18].

### Statistical Analysis

All the data were recorded in Excel (Microsoft Corporation) and assessed for accuracy [19]. The statistical analysis was completed using SPSS, version 25 (IBM Corporation) [20]. Descriptive statistics were obtained to understand the characteristics of the data. Statistically, to understand the concurrent validity of the FCV-19S with the PHQ-9 and GAD-7, the Pearson correlation was used. Furthermore, to understand the impact of the students’ sociodemographic characteristics on these scales, multiple linear regression was used.

## Results

### Sociodemographic Characteristics

The sociodemographic characteristics of the participants are summarized in Table 2. Among the 324 respondents, slightly more male students (180, 55.6%) participated than female students (144, 44.4%). Of the 324 participants, most were in the age group of 18-21 years (190, 58.6%), and 256 (79%) were enrolled in bachelor’s degree programs. In addition, 37/324 participants (11.4%) reported that one of their family members had become infected with COVID-19, which seems to a be very low percentage compared with the recent spread of COVID-19 among the urban population.

**Table 2:** Sociodemographic characteristics of the study participants (N=324).

Sociodemographic characteristic		Value, n (%)
**Age (years)**		
	18-21	190 (58.6)
	22-25	116 (35.8)
	26-29	8 (2.5)
	≥30	10 (3.1)
**Gender**		
	Male	180 (55.6)
	Female	144 (44.4)
**Degree enrolled**		
	Diploma	11 (3.4)
	Bachelor’s degree	256 (79)
	Master’s degree	53 (16.4)
	PhD	4 (1.2)
**Family member infected with COVID-19**		
	Yes	37 (11.4)
	No	287 (88.6)

### Psychometric Scales

Descriptive statistics were studied for all three psychometric scales to understand the impact of the COVID-19 pandemic on the mental health of the students who participated in this study. The median scores of the FCV-19S, PHQ-9, and GAD-7 were found to be 22 (range 17-28), 5.5 (range 2-10.75), and 5 (range 0-9), respectively. The magnitudes of COVID-19 fear, symptoms of depression, and anxiety were graded according to their cutoff scores, as shown in Table 3. This study shows an alarming picture of the impact of the COVID-19 pandemic on the mental health of students, with 223 of 324 students (68.8%) having high fear of COVID-19, 93 students (28.7%) having moderate to severe depression, and 167 students (51.5%) having mild to severe GAD.

**Table 3:** Categorization of the severity of fear of COVID-19, anxiety, and depression among the participating students according to their scale cutoff scores (N=324).

Symptoms and severity		Value, n (%)
**Fear of COVID-19**		
	High	223 (68.8)
	Low	101 (31.2)
**Depression**		
	Minimal	142 (43.8)
	Mild	89 (27.5)
	Moderate	46 (14.2)
	Moderately severe	33 (10.2)
	Severe	14 (4.3)
**Anxiety**		
	Minimal	157 (48.5)
	Mild	90 (27.8)
	Moderate	38 (11.7)
	Severe	39 (12)

### Impact of Risk Factors on the Psychometric Scales

As revealed in Table 4, after multiple linear regression, we found no impact of any of the identified risk factors on the FCV-19S score. However, the table shows that having a family member infected with COVID-19 is significantly associated with GAD-7 and PHQ-9 scores, respectively. If any family member of a college student became infected with COVID-19, their GAD-7 and PHQ-9 scores increased by 2.4 and 3.6, respectively.

**Table 4:** Impact of sociodemographic characteristics on the psychometric scale scores. Multiple linear regression is statistically significant at *P*<.05.

Sociodemographic characteristics		FCV-19S^a,b^		PHQ-9^c,d^		GAD-7^e,f^	
		B (95% CI)	*P* value	B (95% CI)	*P* value	B (95% CI)	*P* value
Constant		23.464 (22.229-24.699)	<.001	6.5 (5.372 to 7.628)	<.001	5.54 (4.454 to 6.626)	<.001
**Age^g^ (years)**							
	22-25	–1.665 (–3.392 to 0.063)	.06	–0.343 (–1.921 to 1.235)	.67	–0.087 (–1.606 to 1.432)	.91
	26-29	–4.435 (–9.787 to 0.916)	.10	–0.373 (–5.261 to 4.516)	.88	0.394 (–4.311 to 5.099)	.87
	≥30	1.3 (–4.004 to 6.605)	.63	–2.739 (–7.584 to 2.107)	.27	0.618 (–4.046 to 5.282)	.80
**Sex^h^**							
	Female	0.258 (–1.251 to 1.767)	.74	1.145 (–0.234 to 2.523)	.10	0.806 (–0.521 to 2.133)	.23
**Degree enrolled^i^**							
	Master’s	–1.288 (–3.685 to 1.109)	.29	–1.071 (–3.26 to 1.119)	.34	–0.364 (–2.472 to 1.743)	.73
	PhD	–7.294 (–15.299 to 0.711)	.07	–4.098 (–11.41 to 3.215)	.27	–5.123 (–12.162 to 1.915)	.15
	Diploma	–1.818 (–5.930 to 2.294)	.385	–2.548 (–6.304 to 1.208)	.18	–2.181 (–5.796 to 1.435)	.24
**Family member infected with COVID-19^j^**							
	Yes	–0.659 (–3.016 to 1.699)	.58	3.689 (1.536 to 5.843)	.001	2.474 (0.401-4.546)	.02

^a^FCV-19S: Fear of COVID-19 Scale.

^b^*R*^2^=0.050.

^c^PHQ-9: Patient Health Questionnaire-9.

^d^*R*^2^=0.065.

^e^GAD-7: Generalized Anxiety Disorder-7 Scale.

^f^*R*^2^=0.033.

^g^Reference category for the independent variable of age: 18-21 years.

^h^Reference category for the independent variable of sex: male.

^i^Reference category for the independent variable of degree enrolled: bachelor’s degree.

^j^Reference category for the independent variable of family member infected with COVID-19: no.

### Participants’ Responses to the Psychometric Scales

#### FCV-19S Responses

In the survey, 207/324 respondents (63.7%) were found to be afraid of COVID-19. Watching COVID-19 stories and reports on social media seemed to have a major effect on the mental health of 183/324 students (56.3%), making them nervous or anxious. Among the 324 respondents, 71 (21.8%) stated that when they thought about COVID-19, they could not sleep properly due to fear of the disease, and 176 (57.2%) said they were uneasy when thinking about it. On the other hand, 128/324 students (39.3%) said that their hands did not become clammy and their heart did not race when thinking of COVID-19.

#### PHQ-9 Responses

Of the 324 students, 202 (62.2%) said they had no motivation or enjoyment when participating in activities, and 160 students (49.3%) reported feeling down, sad, or hopeless. Moreover, 162/324 respondents (50%) appeared to have difficulty falling asleep, slept for a long time, or slept too much, and they found it difficult to focus on activities such as reading or watching television. Of the 324 respondents, 92 (28.4%) had thoughts of being “better off dead” or hurting themselves.

#### GAD-7 Responses

Of the 324 students, 134 (41.3%) felt that they had become restless and irritable, and 169 students (52%) were afraid that something awful would happen to them for several days; 137 students (42.2%) agreed that they felt anxious, whereas 194 students (59.8%) worried too much.

### Concurrent Validation of the FCV-19S

The FCV-19S was significantly associated with the GAD-7 and PHQ-9, respectively. The Pearson correlations of the FCV-19S with the PHQ-9 and GAD-7 were found to be *r=*0.474 and *r=*0.492, respectively (both *P*<.001; correlation was statistically significant at a *P* value of <.01 [2-tailed]). This moderately positive correlation of the FCV-19S scale with the PHQ-9 scale and GAD-7 scale helps to predict that an increase in fear of COVID-19 will ultimately increase the anxiety and depressive symptoms in students [21]. Therefore, the FCV-19S can give an overall idea of the levels of fear, depression, and anxiety among students caused by the COVID-19 pandemic.

## Discussion

### Principal Findings

This study aims to understand the impact of the COVID-19 pandemic on the mental health of students and to identify the risk factors that magnify mental health disorders in the current situation. This survey revealed a high prevalence of self-reported anxiety, depression, and fear of COVID-19 among college students in India. Among the risk factors, a family member contracting COVID-19 significantly increased the students’ levels of anxiety and depression. This study also found a moderate positive correlation of the FCV-19S with the GAD-7 and PHQ-9, respectively.

In this study, we found that 223 of 324 students (68.7%) had high fear of COVID-19, which is almost double the proportion found by Parlapani et al [14] among general people in Greece (ie, 35.7%) but was consistent with that in a study by Gritsenko et al [22] that was conducted among Russian and Belarusian university students. This drastic difference in the fear of COVID-19 between students in India and the general public in Greece may be due to differences in the region, population category, and upsurge of COVID-19 cases, along with the government’s effective measures to address mental health disorders among its residents. A study conducted in India by Sathe et al [23] reported that a moderate to severe level of fear of COVID-19 is prevalent in 49% of the general population, and Doshi et al [24] found that 48% of the general public in India indicated being afraid of COVID-19, which is a lower percentage compared to that of the college students in this study. Elemo et al [25] found average scores of 19.99 (SD 6.6) on the FCV-19S among international students in Turkey, which indicates a higher degree of anxiety due to COVID-19 [1]. These studies reveal a higher degree of fear of COVID-19 among students than in the general public in many countries.

The 2016 National Mental Health Survey reported a 2.7% prevalence of depressive disorder and a 3.1% prevalence of anxiety in the Indian population; however, in this study, we found alarming upsurges in the levels of anxiety and depression, mainly due to the COVID-19 pandemic [26]. It was found that 167 of 324 students (51.5%) had mild to severe anxiety symptoms, and 93 students (28.7%) had moderate to severe depressive symptoms. The results of this study were consistent with those of a study by Rehman et al [27], which was conducted during the first wave of the COVID-19 pandemic among students in India; however, the level of anxiety found in this study was lower than that in a study of college students in the United States, where 71% believed that their anxiety was increased by the COVID-19 outbreak [28]. A study conducted in Malaysia among university students found that anxiety was prevalent in 29% of the students, which is a lower percentage compared to the findings of this study [29]. When comparing our results with those of a survey conducted among university students in Bangladesh [30], the levels of anxiety were similar, but the level of depression was higher in the other study.

In contrast, a study conducted by Shah et al [31] among the global population to understand the impact of the COVID-19 pandemic on mental health found that 47% of students had depression due to the COVID-19 pandemic, but they had a similar level of anxiety compared to that in this study. A survey conducted by Aftab et al [32] among undergraduate and postgraduate students studying medicine worldwide found a prevalence of depression of 41.5% in these students, which is dramatically higher than that found this study; however, anxiety among those students was less prevalent than among those in this study. These studies reveal a higher degree of anxiety among college students during the COVID-19 outbreak, and web-based learning is an important cause of increased anxiety and depression [33-35].

Multiple linear regression showed that among the identified risk factors, infection of a family member with COVID-19 had a significant impact on anxiety and depression among students; however, there was no impact of any identified risk factor on the fear of COVID-19. These findings are consistent with studies conducted in India to assess fear of COVID-19, anxiety, and depression levels, as the same guidelines were implemented for the COVID-19 pandemic across the country [24,27]. Compared with international students, the findings of this study were consistent with the results of Islam et al [3], who conducted a study among university students in Bangladesh and reported no impact of age or gender on anxiety or depression levels, respectively; however, a study conducted among university students in France [30] reported an effect of gender on anxiety and depression. The multinational study conducted by Pramukti et al [36] among university students to understand the impact of the COVID-19 pandemic on anxiety and suicidal thoughts found a significant impact of gender on symptoms of anxiety, with a *P* value <.001; this is not consistent with the findings of this study [36]. However, a study conducted among university students in Malaysia [32] found no impact of age or gender on anxiety symptoms, which is similar to the findings of this study. The review of these studies reveals that the effects of sociodemographic factors on anxiety and depression differ according to the country and region.

The FCV-19S is a recently developed tool to understand the fear caused by the COVID-19 pandemic among the public. Various versions of this scale have been validated with other established psychometric scales used to understand anxiety, depression, stress, etc. This study also validated the English version of the FCV-19S with the GAD-7 and PHQ-9, respectively. The findings of this study are consistent with the concurrent validity of the Greek version of the FCV-19S with the PHQ-9, which reports a moderate positive correlation; however, our findings are inconsistent with the concurrent validity of the Greek version of the FCV-19S with the GAD-7 [21,37]. The Spanish version of the FCV-19S scale showed a weak positive correlation with the GAD-7 and PHQ-9 among males, whereas among females, it showed a moderate positive association with GAD-7 and a weak positive correlation with the PHQ-9 [38]. The validity of the Japanese version of the FCV-19S with the GAD-7 was consistent with the findings of this study [39]. These studies reveal that the strength of the association differs when a different version of the FCV-19S is used.

### Conclusion

This research indicates that fear of COVID-19 is very high among Indian students, along with anxiety and depression. Furthermore, among the identified risk factors, having a family member infected by COVID-19 significantly impacted anxiety and depression among students. This study also concludes that the FCV-19S has a moderate positive correlation with the GAD-7 and PHQ-9, respectively.

To mitigate the fear, anxiety, and depression caused by the COVID-19 pandemic, students should be encouraged to pursue healthier lifestyles during the pandemic. We also recommend developing and implementing various policies at the government level to reduce the effects of the COVID-19 pandemic on mental health.

### Study Limitations

Some constraints are included in this report. First, there is an unequal distribution of respondents in this sample, and because it is a cross-sectional study, casual intervention cannot be performed. Second, there are very few diploma students; therefore, the survey results cannot be generalized to the whole student population. Third, the questionnaire was self-administered, so it is difficult to understand whether it was reasonably completed (ie, social desirability bias and semblance). Fourth, because the survey was internet-based, the study did not actively collect the responses of learners who are not linked to social media. Finally, this study adopted a cross-sectional study design; therefore, cause and effect relationships cannot be established.

## Abbreviations

CHERRIES: Checklist for Reporting Results of Internet E-Surveys
FCV-19S: Fear of COVID-19 Scale
GAD: generalized anxiety disorder
GAD-7: Generalized Anxiety Disorder-7 scale
PHQ-9: Patient Health Questionnaire-9
SARS: severe acute respiratory syndrome
STROBE: Strengthening the Reporting of Observational Studies in Epidemiology
